# On the Internal Administration of Chloroform in Pernicious Fever

**Published:** 1867-10

**Authors:** Sampson Eagon

**Affiliations:** Marshall, Texas


### SELECTIONS.

---

### *On the Internal Administration of Chloroform in Pernicious Fever.* By Sampson Eagon, M. D., Marshall, Texas.

The object of this brief paper is to present to the Profession, the clinical history of six cases of Pernicious Fever, in the treatment of which Chloroform internally administered, was relied upon as the principal therapeutic means. These cases embrace in their number, one or more of the several recognized varieties of the disease, and are as follows:

1st, one of the cerebral variety; 2d, three of the abdominal variety; 3d, one of the thoracic variety; 4th, one of the necreamal variety.

The first case, occurring in the vicinity of Opelousas, La., in the Fall of '61, the remainder occurring in the neighborhood of this place, (Marshall Texas,) during the last Summer and Autumn.—Beginning seriatim, with these cases, the first (cerebral) was of moderate reverity, though possessing in a well pronounced degree, all the distinctive features of the disease. The patient, a negro man, æt. about 25, with first paroxysm, was seen two hours after the accession of the chill, when chloroform f. ʒj. was administered in double that quantity of glycerine, as an excipient; synapisms the meanwhile were applied to the lower extremities. The good effects of this dose were experienced in less than ten minutes, as indicated by the diminished frequency and augmented force of the hearts action, by returning warmth to the extremities, together with almost complete subsidence of delirium; in fifteen minutes the dose of chloroform was repeated, and in half an hour re-action was completely re-established. The patient was now put upon the liberal use of quinia, which was continued for four or five days; convalescence rapid; no recurrence.

The second case, (belonging to the abdominal variety) the subject, a young lady, æt. 17, was of much more gravity than that just detailed, the cold stage having existed for three hours with unabated violence, no efficient means having yet been resorted to for relief. Treatment, chloroform f. ʒj. in glycerine, every fifteen minutes till three drachms have been taken; active revulsion to the spine and extremities by means of symapisms and friction with hot oil of terpentine. Reäction was thus brought about in an hour, and the patient fell into a quiet slumber, from which she awoke in four or five hours, quite relieved. After treatment, quinia continued as in the other cases; no recurrence.

The second and third abdominal cases were so similar in their character, mode of treatment, and termination, as to admit of a common description. Both cases occurred in young adult males; were of great severity; were not seen by the physician for three or four hours after the commencement of the chill, which was continuing to deepen into a collapse. In these cases there were frequent, though not copious, discharges of sero-sanguinolent matter. The patient, indeed, presented very much the aspect of one laboring under epidemic cholera, in the stage of collapse. Treatment, chloroform f. ʒ i. in gycerine. A favorable impression made upon the patients in fifteen minutes, remedy repeated, at this interval till four drachms had been given. Revellents to the spine and extremities vigorously employed. Reäction complete in an hour and a half, favored by slight febrile movement, which lasted for an hour or more, the patients, the meanwhile, falling into a quiet sleep, from which they awoke in six or eight hours relieved. After treatment, quinia, &c.; convalescence favorable; no recurrence of paroxysm.

The fifth case was thoracic, presenting the ordinary constitutional symptoms of this stadium of the disease (cold stage.) superadded to which was, laborious oppression of the respiratory organs, as shown by the much accellerated and difficult respiration. The variety of the disease is justly regarded as second only in gravity of import, to the almost necessarily fatal necreamial variety. Chill had lasted three hours before treatment was begun. Treatment the same as employed in the preceding cases, with like effects; convalescence protracted. After treatment quinia, &c.; no recurrence.

The sixth and last case (necreamial) was one of overwhelming severity, the nervous system seeming to have been almost paralized, whilst its dependencies seemed to suffer in like degree, from the poisonous effects of the malarial principle. The blood, indeed, upon which every organ and tissue of the body is dependent for its vital activity, seemed to be so changed and vitiated in quality, as to no longer afford appropriate material for nutrition. The subject in this case was a German, æt. about 30, of a well developed and originally vigorous frame, but at the time of attack, much reduced in strength from the protracted intemperate use of alcoholic beverages. Patient seen not until twelve hours had elapsed from the supervention of the chill. Treatment, the same as in the preceding cases, with the exception that the remedy was given rather more liberally, and persevered in for a longer period. No impression favorable or otherwise seemed to be produced by chloroform, or any of the various remedies resorted to in the case. The patient died in six hours from the time I first saw him.

In treating of this affection, I have preferred to employ the classification adopted for the practical reason, that whilst the disease is essentially the same in all its various forms, requiring the same general mode of management, yet in reference to the diagnosis, it is important to bear in mind its several varieties, when there will be generally little difficulty in referring each particular case to the class to which it belongs.

My attention was first directed to chloroform administered internally, in 1860, by the eulogies pronounced upon its virtues in three cases, by the late Prof. E. D. Fenner, of New Orleans, who had prior to that period employed it with very gratifying results in several cases, as well as in the cold stage of ordinary intermittents, with like results. Dr. F. remarked on this occasion, that, "from the very prompt and happy effects experienced in the limited number of cases, in which I have seen the remedy exhibited, I am inclined to regard chloroform, internally administered, in the dose of from half a drachm to a drachm, repeated at the interval of fifteen or twenty minutes, as possessing more potency than any other article of the materia medica, in bringing about reäction from the frightful collapse of congestive fever. Having resided and practiced my profession since '60, in a highly malarial district, an ample opportunity has presented itself, which has been embraced, of putting to the test Dr. F.'s assertion as to the virtue of chloroform in relieving a paroxism of intermittent fever in the cold stage, I am happy to be able to add, that in doing so I have had occasion, in more instances than one to feel gratefully thankful to this distinguished gentleman for his valuable suggestion. Several articles have recently appeared in the different Medical Journals of this country, each bearing testimony to the value of chloroform in ordinary intermittents, given in the first stage. My own experience accords well with the views expressed in those articles. I regard the disease, (as I believe most Southern physicians do,) pernicious and intermittent fever, as differing in degree only, and not in kind. The treatment of both, therefore, is rationally conducted upon the same general principles.

As to the modus operandi of chloroform in relieving the cold stage of intermittent fever, I have seen no explanation. The most plausible hypothesis of its mode of action, it seems to me, is, that a powerfully stimulating impression is produced upon the stomach by immediate contact of the remedy, which impression is rapidly conveyed chiefly through the medium of the nervous system to the capillaries, exciting, in this system of vessels, remote sympathy. As to the prophylactic, or antiperiodic power of chloroform in intermittent fever, I am inclined to think it possesses no greater virtue in this way, than is common to all narcotics.—*Richmond Medical Journal.*

